# Production of (*R*)-mandelic acid from styrene, *L-*phenylalanine, glycerol, or glucose via cascade biotransformations

**DOI:** 10.1186/s40643-021-00374-6

**Published:** 2021-03-04

**Authors:** Benedict Ryan Lukito, Zilong Wang, Balaji Sundara Sekar, Zhi Li

**Affiliations:** 1 Department of Chemical and Biomolecular Engineering, National University of Singapore, Singapore, 117585 Singapore; 2 Synthetic Biology for Clinical and Technological Innovation (SynCTI), Life Sciences Institute, National University of Singapore, Singapore, 117456 Singapore

**Keywords:** Sustainable synthesis, Cascade reaction, Biotransformation, Mandelic acid, Renewable feedstocks, Coupled-cells biotransformation, *L-*phenylalanine, Styrene

## Abstract

(*R*)*-*mandelic acid is an industrially important chemical, especially used for producing antibiotics. Its chemical synthesis often uses highly toxic cyanide to produce its racemic form, followed by kinetic resolution with 50% maximum yield. Here we report a green and sustainable biocatalytic method for producing (*R*)-mandelic acid from easily available styrene, biobased *L-*phenylalanine, and renewable feedstocks such as glycerol and glucose, respectively. An epoxidation-hydrolysis-double oxidation artificial enzyme cascade was developed to produce (*R*)-mandelic acid at 1.52 g/L from styrene with > 99% *ee*. Incorporation of deamination and decarboxylation into the above cascade enables direct conversion of *L-*phenylalanine to (*R*)-mandelic acid at 913 mg/L and > 99% *ee*. Expressing the five-enzyme cascade in an *L*-phenylalanine-overproducing *E. coli* NST74 strain led to the direct synthesis of (*R*)-mandelic acid from glycerol or glucose, affording 228 or 152 mg/L product via fermentation. Moreover, coupling of *E. coli* cells expressing *L*-phenylalanine biosynthesis pathway with *E. coli* cells expressing the artificial enzyme cascade enabled the production of 760 or 455 mg/L (*R*)-mandelic acid from glycerol or glucose. These simple, safe, and green methods show great potential in producing (*R*)-mandelic acid from renewable feedstocks.

**Supplementary Information:**

The online version contains supplementary material available at 10.1186/s40643-021-00374-6.

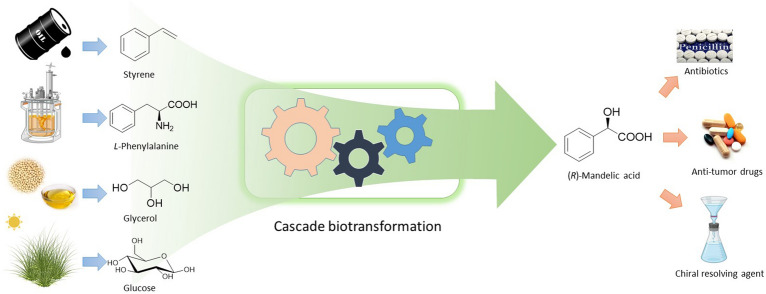

## Introduction

Biosynthesis of high-value chemicals from renewable feedstocks for sustainable manufacturing of chemicals has received an increasing attention in attribution to the change of the global climate and oil depletion (Biermann et al. 2011; Christensen et al. 2008; Tuck et al. 2012; Vennestrøm et al. 2011). Biocatalyst also has been proven to be a useful tool for the synthesis of high-value chemicals due to its high chem-, regio-, and stereoselectivity (Bornscheuer et al. 2012; Dong et al. 2018; Gandomkar et al. 2019; Reetz 2013; Schrittwieser et al. 2018; Winkler et al. 2018; Wu and Li 2018; Wu et al. 2020). The replacement of the chemical synthesis with clean, green, and high-yielding biocatalytic preparation is considered as one of the most attractive strategies to realize the sustainable manufacturing of chemicals.

(*R*)-mandelic acid (MA) is an important and useful chiral molecule for the production of antibiotics such as cephalosporins, semi-synthetic penicillins (Bhatia et al. 2014; Jiang et al. 2016; Zhang et al. 2010), as well as anti-tumor (Surivet and Vatèle 1999) and anti-obesity agents (Mills et al. 1983). It is also intensively applied in the resolution of both racemic amines (Kinbara et al. 1996) and alcohols (Whitesell and Reynolds 1983) as the chiral resolving agent. In addition, (*R*)*-*MA was used as a chiral template to synthesize chirally imprinted mesoporous materials (Yutthalekha et al. 2015).

(*R*)*-*MA is mainly synthesized chemically. The cyanide-based method involved two-step reactions including cyanation of benzaldehyde using either NaCN or transition metal catalysts, such as titanium or vanadium complexes of chiral ligands, followed by the hydrolysis of mandelonitrile using HCl to give enantiopure (*R*)*-*MA (Blacker and Houson 2002; Corson et al. 2003). This method requires the use of highly toxic cyanide and expensive transition metal catalyst together with chiral ligands but gives unsatisfied *ee*, low overall yields, and generates a lot of by-products and large amount of waste. The dichloroacetophenone-based method involved the chlorination of acetophenone with chlorine, followed by alkaline hydrolysis by NaOH at 65 °C and the acidolysis with HCl. This method requires the use of toxic and dangerous Cl_2_, high temperature, and also suffers from side products problems (mono, tri-choloroacetophenone) (Aston et al. 2003). Practically, chemical methods are mainly used to produce racemic MA. Therefore, the kinetic resolution is often required for obtaining enantiopure (*R*)- or (*S*)-MA, but the theoretical maximum yield is only 50%.

Recently, several methods for biosynthesis of (*R*)-MA are reported. The single step biosynthesis of (*R*)-MA is mainly based on the kinetic resolution of racemic mandelonitrile with nitrilases (NLases) and the kinetic resolution of racemic MA using enzymes such as, lipases, esterases, or dehydrogenases (Chen et al. 2017; Detzel et al. 2011; He et al. 2007; Jiang et al. 2017, 2016; Li et al. 2007; Wang et al. 2015; Xiao et al. 2005). These methods usually suffer from low efficiency and low yield, and the racemic feedstocks are still derived from chemical synthesis. The bioreduction of phenylglyoxylic acid (PGA) to produce (*R*)-MA using alcohol dehydrogenase is also reported (Jiang et al. 2016; Xiao et al. 2005). The synthesis of (*R*)*-*MA via two-step reactions involving the asymmetric reduction of ethyl benzoylformate catalysed by *Saccharomyces cerevisiae* resting cells, followed by the subsequent hydrolysis with *Bacillus cereus* resting cells is also reported (Zhimin et al. 2017). Nevertheless, this approach involved the use of extremely high cell density of yeast (100 g cell dry weight/L, cdw/L) and *Bacillus cereus* (80 g cdw/L), and the chemical production of substrates still requires the use of toxic reagents.

Fermentative production of bio-based chemicals from sugars have received increasing attention (Becker and Wittmann 2015; Keasling 2010; Lee et al. 2012; Nielsen and Keasling 2016; Woolston et al. 2013). An engineered *E. coli* strain containing a pathway involving the hydroxymandelate synthase produced 680 mg/L (*R*)-MA from glucose (Sun et al. 2011). Recently, biosynthesis of MA from glucose in *Saccharomyces cerevisiae *via a natural pathway containing a hydroxymandelate synthase was achieved at 236 mg/L (Reifenrath and Boles 2018). Nevertheless, the titer of the fermentative production of (*R*)-MA from glucose still needs improvement, and new and efficient pathways are highly wanted.

**Scheme 1:**
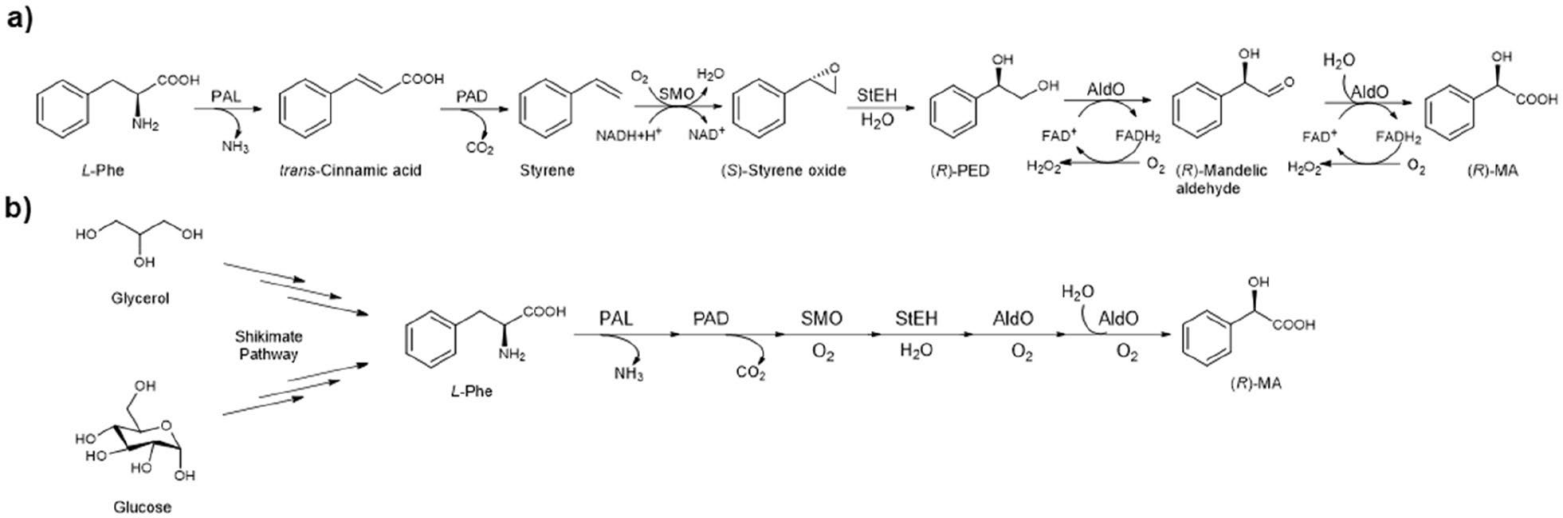
Enzyme cascades for the bioproduction of (*R*)-mandelic acid from *L*-Phe, glucose or glycerol. **a** The five-enzyme artificial cascade containing PAL, PAD, SMO, StEH, and AldO for the conversion of *L*-Phe to (*R*)-MA. **b** Biotransformation of glycerol or glucose to (*R*)-MA by combining *L*-Phe biosynthesis pathway with five-enzyme artificial cascade

**Fig. 1:**
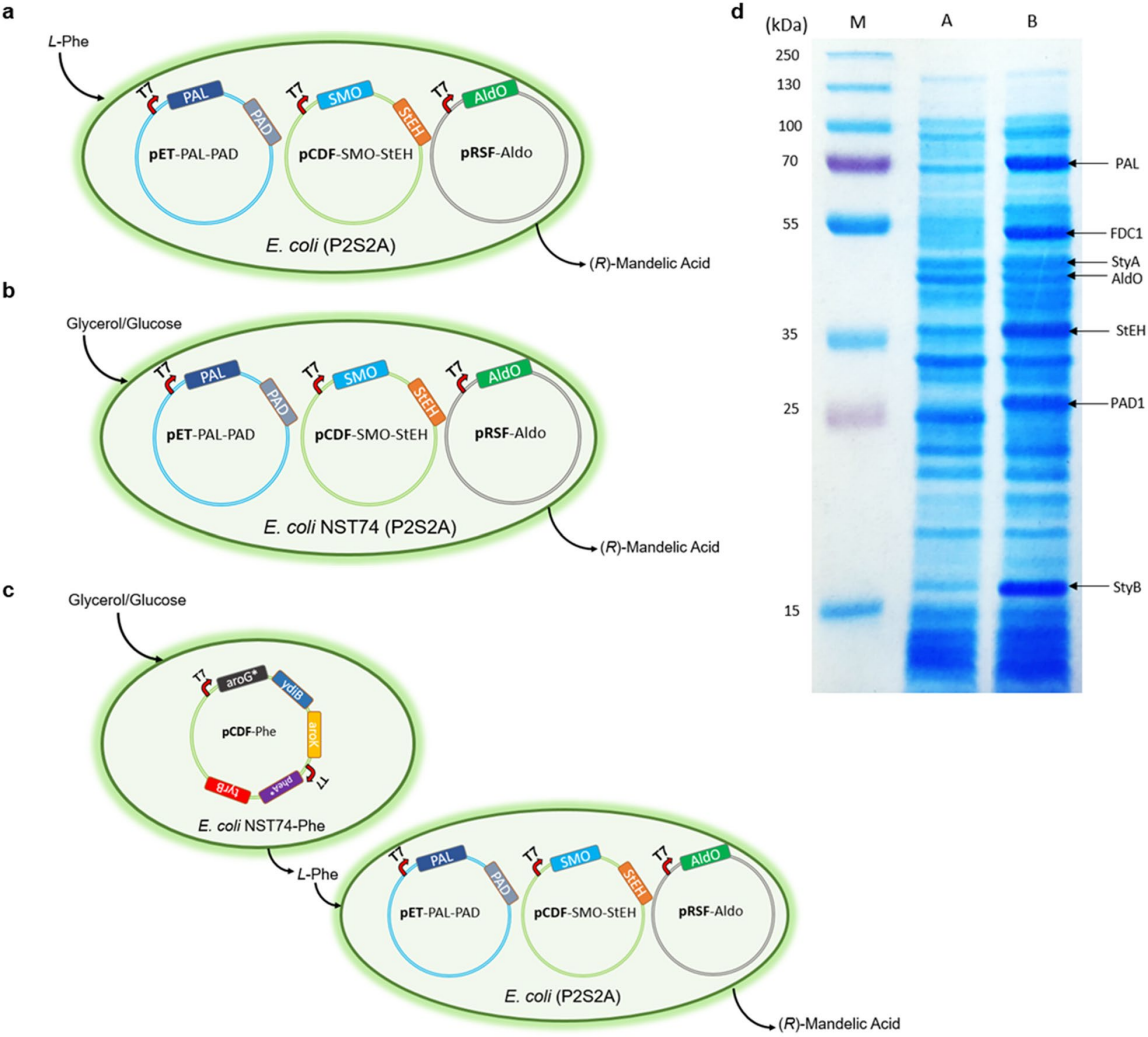
Construction of *E. coli* cells co-expressing multiple enzymes for the bioproduction of (*R*)-MA. **a** *E. coli* (P2S2A) strain harbouring plasmid pET-PAL-PAD encoding PAL and PAD, plasmid pCDF-SMO-StEH encoding SMO and StEH, and plasmid pRSF-AldO encoding AldO for the conversion of *L-*phenylalanine to (*R*)-MA. **b** *E. coli* NST74 (P2S2A) strain harbouring plasmid pET-PAL-PAD encoding PAL and PAD, plasmid pCDF-SMO-StEH encoding SMO and StEH, and plasmid pRSF-AldO encoding AldO for the conversion of glycerol or glucose to (*R*)-MA. **c** Coupled *E. coli* NST74-Phe strain over-expressing five limiting enzymes in Shikimate pathway with *E. coli* (P2S2A) expressing five-enzyme artificial cascade for the conversion of glycerol or glucose to (*R*)*-*MA. **d** SDS-PAGE. Lane M: protein marker; Lane A: total intracellular protein of *E. coli* NST74; Lane B: total intracellular protein of *E. coli* NST74 (P2S2A)

Our interest is to develop artificial enzyme cascades to produce (*R*)-MA with enhanced productivity from simple, cheap, or renewable feedstocks, such as styrene, *L*-phenylalanine (*L*-Phe), glycerol or glucose (Scheme 1). Previously, we successfully developed styrene-involved pathways to produce several non-natural high-value chemicals (Lukito et al. 2019a, 2019b; Wu et al. 2017; Zhou et al. 2016) in high yield and high *ee*. Here we report the development of novel artificial enzyme cascades for (*R*)-MA production from cheap and easily available styrene, bio-based *L*-Phe (Fig. 1a), as well as the combination of natural *L*-Phe biosynthesis pathway and the artificial enzyme cascade for the production of (*R*)-MA from glucose or glycerol in single strain (Fig. 1b) and coupled cells biotransformation (Fig. 1c), respectively. These results successfully demonstrated the enhancement and synthetic potential for producing (*R*)-MA from the cheap and easily available renewable feedstocks.

## Materials and methods

### Genetic engineering of recombinant *E. coli* strains

The gene of alditol oxidase (AldO) from *Streptomyces coelicolor* A3(2) was codon optimized and synthesized based on the reported gene sequence (GenBank accession No. SCO6147) by GenScript. The gene fragments of AldO was amplified by polymerase chain reaction (PCR) using Phusion DNA polymerase with forward primer 5′-TATATCATGAGCGACATTACCGTTACCAAC-3′ and reverse primer 5′-CTCGAATTCTTAACCTGCCAGAACGCCACGAAC-3′ (restriction sites are underlined). The PCR products were digested with *Bsp*HI and *Eco*RI and then ligated with *Nco*I and *Eco*RI digested pRSFDuet-1 to give pRSF-AldO. *E. coli* (AldO) strain was made by transforming pRSF-AldO into chemical competent *E. coli* T7 cells.

*E. coli* (S2A) strain was made by co-transformation of pRSF-AldO and previously constructed pCDF-SMO-StEH (Zhou et al. 2016) into chemical competent *E. coli* T7 cells. *E. coli* (P2S2A) strain was made by transforming previously constructed pET-PAL-PAD (Zhou et al. 2016) into chemical competent *E. coli* (S2A) cells. The engineering of *E. coli* NST74 (P2S2A) strain was similar to above mentioned procedures using our previously constructed *E. coli* NST74 (DE3) cells (Zhou et al. 2018). All the strains and enzymes used in this study are listed in Additional file 1: Table S1 and Table S2 in Supporting Information.

### Biotransformation of (*R*)-1,2-phenylethanediol to (*R*)-mandelic acid with resting cells of *E. coli* (AldO) in aqueous phase

*E. coli* (AldO) cells were cultured, harvested, and resuspended in 10 mL of KP buffer (potassium phosphate buffer, 100–200 mM, pH 8.0) containing 2.76 g/L (20 mM) of (*R*)-1,2-phenylethanediol (PED) to a cell density of 10–15 g cdw/L to perform the biotransformation at 30 °C and 250 rpm for 24 h. 50 µL of the aqueous phase was collected and diluted with 450 µL ultrapure water containing 0.5% trifluoroacetic acid (TFA) and 500 µL acetonitrile (ACN) containing 2 mM benzyl alcohol. The samples were analysed using HPLC to quantify the concentration of (*R*)*-*MA and (*R*)*-*PED.

### Biotransformation of styrene to (*R*)-mandelic acid with resting cells of *E. coli* (S2A) in two-phase system

*E. coli* (S2A) cells were harvested and resuspended in 10 mL of KP buffer (200 mM, pH 8.0) containing 0.5% glucose to a cell density of 15 g cdw/L. 10 mL of *n-*hexadecane containing 2.08 g/L (20 mM) of styrene was added to perform the biotransformation at 30 °C and 250 rpm for 24 h. 50 µL of the aqueous phase was collected and diluted with 450 µL ultrapure water containing 0.5% TFA and 500 µL ACN containing 2 mM benzyl alcohol. The samples were analysed using HPLC to quantify the concentration of (*R*)*-*MA and (*R*)*-*PED. 50 µL of the organic phase was collected and diluted with 950 µL ethyl acetate containing 2 mM benzyl alcohol, and the samples were analysed using GC to quantify the concentration of styrene and (*S*)-styrene oxide.

### Biotransformation of *L*-phenylalanine to (*R*)-mandelic acid with resting cells of *E. coli* (P2S2A) in two-phase system

*E. coli* (P2S2A) cells were harvested and resuspended in 10 mL of KP buffer (200 mM, pH 8.0) containing 0.5% glucose and 2.48 g/L (15 mM) of *L*-Phe to a cell density of 15 g cdw/L. 10 mL of *n-*hexadecane was added to perform the biotransformation at 30 °C and 250 rpm for 24 h. 50 µL of the aqueous phase was collected and diluted with 450 µL ultrapure water containing 0.5% TFA and 500 µL ACN containing 2 mM benzyl alcohol. The samples were analysed using HPLC to quantify the concentration of (*R*)*-*MA, (*R*)*-*PED, and *L*-Phe. 50 µL of the organic phase was collected and diluted with 950 µL ethyl acetate containing 2 mM benzyl alcohol, and the samples were analysed using GC to quantify the concentration of styrene and (*S*)-styrene oxide.

### Fermentative production of (*R*)-mandelic acid from glycerol or glucose with single *E. coli* strain in aqueous system

*E. coli* NST74 (P2S2A) strains were inoculated with LB medium containing appropriate antibiotics (50 µg/mL kanamycin, 50 µg/mL streptomycin, and 100 µg/mL ampicillin) at 37 °C and 250 rpm for 8–10 h. The inoculated strain was subsequently transferred and grown into a 250 mL-baffled culture flask with M9 or TB medium and appropriate antibiotics in a total volume of 50 mL. IPTG was added to a final concentration of 0.1 mM to induce the protein expression when OD600_nm_ reached to ~ 0.6. The cells were continued to grow at 25 °C for another 24 h. Cells were then harvested after 24 h of growth by centrifugation (4000* g*, 10 min). Product concentration was subsequently measured by HPLC.

### Fermentative production of (*R*)-mandelic acid from glycerol or glucose with single *E. coli* strain in *n-*hexadecane-aqueous two-phase system

*E. coli* NST74 (P2S2A) strains were inoculated with LB medium containing appropriate antibiotics (50 µg/mL kanamycin, 50 µg/mL streptomycin, and 100 µg/mL ampicillin) at 37 °C and 250 rpm for 8–10 h. The inoculated strain was subsequently transferred and grown in a 250 mL-baffled culture flask containing M9 or TB medium with appropriate antibiotics in a total volume of 50 mL. When OD600_nm_ reached to ~ 0.6, IPTG was added at a final concentration of 0.1 mM to induce the protein expression, together with 25 mL of *n-*hexadecane. The cells continued to grow at 25 °C for another 24 h. Product concentration in culture media was subsequently measured by HPLC.

### Bioproduction of (*R*)-mandelic acid from glycerol or glucose via coupling two *E. coli* strains in *n-*hexadecane-aqueous two-phase system

Firstly, *L*-Phe was produced from glycerol or glucose by fermentation with *E. coli* NST74-Phe strain (Sekar et al. 2019). Cells were grown in a modified NH_4_-medium containing 10 g/L glycerol or glucose for fermentation, 12.88 g/L (78 mM) and 11.07 g/L (67 mM) of *L-*Phe was obtained from glycerol and glucose at 28 h, respectively. Subsequently, freshly prepared *E. coli* (P2S2A) cells, concentrated KP buffer (pH 8.0), glucose solution and *n*-hexadecane were directly added into the fermentation medium for the conversion of *L*-Phe to (*R*)*-*MA. The final reaction mixture contained 1.65 g/L (10 mM) of *L*-Phe, 200 mM KP buffer (pH 8.0), 1% glucose, 15 g cdw/L of *E. coli* (P2S2A) cells and *n*-hexadecane (1:1, v/v). The biotransformation was performed at 30 °C and 250 rpm for 24 h and product concentration was measured afterwards by HPLC.

## Results and discussion

### Development of artificial enzyme cascade for the conversion of styrene to (*R*)-mandelic acid

**Fig. 2:**
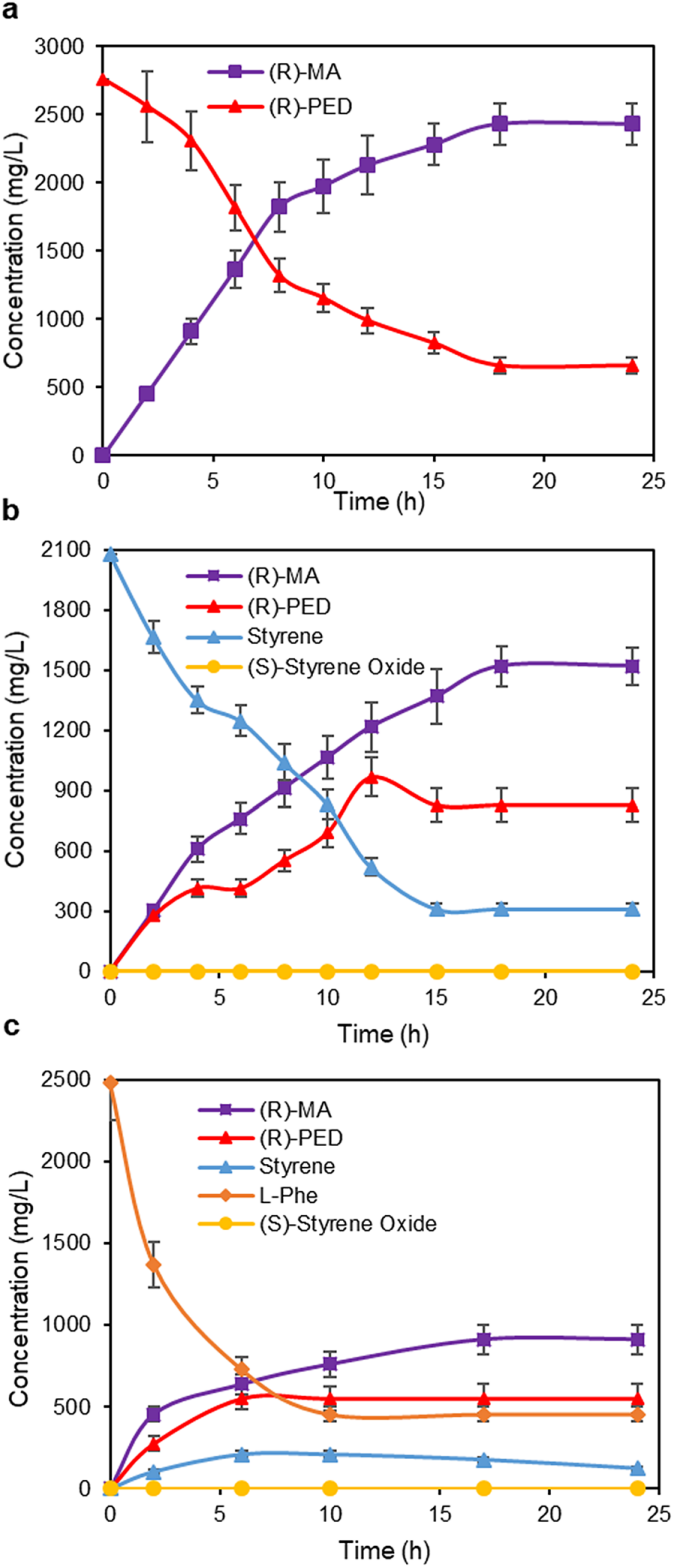
Time course of producing (*R*)-MA from (*R*)-PED, styrene, and *L-*phenylalanine. **a** Biotransformation of (*R*)-PED to (*R*)-MA with *E. coli* (AldO) cells (15 g cdw/L) in KP buffer (200 mM, pH 8.0). **b** Biotransformation of styrene to (*R*)-MA with *E. coli* (S2A) cells (15 g cdw/L) in a mixture of KP buffer and *n-*hexadecane (1:1; v/v). **c** Biotransformation of *L*-Phe to (*R*)-MA with *E. coli* (P2S2A) cells (15 g cdw/L) in a mixture of KP buffer and *n-*hexadecane (1:1; v/v). All reactions were performed at 30 °C and 250 rpm

Previously, a two-enzyme cascade comprising styrene monooxygenase (SMO, consisting of StyA and StyB) from *Pseudomonas* sp. VLB120 and epoxide hydrolase (StEH) from *Solanum tuberosum* was developed for converting styrene to (*R*)-PED. Here, we sought to extend this enzyme cascade to produce (*R*)-MA. Alditol oxidase (AldO) from *Streptomyces coelicolor* A3(2) was chosen for the conversion of (*R*)-PED to (*R*)-MA (Dominic P. H. M. Heuts 2007; van Hellemond et al. 2009). AldO gene was cloned in pRSFDuet-1 plasmid and transformed into *E. coli* T7 strain to give *E. coli* (AldO). Biotransformation of (*R*)-PED to (*R*)-MA was investigated with *E. coli* (AldO) cells (10–15 g cdw/L) in KP buffer system (200 mM, pH 8.0) at 30 °C for 24 h, and 15 g cdw/L of cells gave the best results. The time course is shown in Fig. 2a: (*R*)-MA was produced at a linear rate within the first 8 h with the fast consumption of (*R*)-PED. After 8 h of biotransformation, 1.9 g/L of (*R*)-MA was produced, giving a space–time yield of 237.5 mg/L/h. These results clearly demonstrated AldO catalyzed the conversion of (*R*)-PED to (*R*)-MA. As the reaction continued, the reaction rate gradually dropped down and 2.43 g/L (16 mM) of (*R*)-MA was produced at 24 h in 80% yield, while 600 mg/L (4 mM) of (*R*)-PED remain unreacted. This is probably because of high *K*m (101 mM) toward (*R*)-PED (van Hellemond et al. 2009) and relatively low activity of AldO. Enzyme evolution might be needed for increasing its affinity with (*R*)-PED as well as the activity. Although H_2_O_2_ could be produced during the reaction with 20 mM substrate, the low level of the produced H_2_O_2_ in cells did not influence the enzymatic reactions. This is probably because the *E. coli* cells produced endogenous catalase which could decompose H_2_O_2_. The co-expression of the catalase from *E. coli* with AldO was also tried; however, the product yield was not increased. pRSF-AldO were co-transformed together with pCDF-SMO-StEH to give *E. coli* (S2A) co-expressing SMO, StEH, and AldO for the direct conversion of styrene to (*R*)-MA.

### Bioproduction of (*R*)-mandelic acid from styrene via whole cell biotransformation in n-hexadecane-aqueous two-phase system

Cascade biotransformation of styrene to (*R*)-MA was conducted using *E. coli* (S2A) cells (15 g cdw/L) in a mixture of KP buffer (200 mM, pH 8.0) containing 0.5% glucose and n-hexadecane (1:1; v/v) at 30 °C for 24 h. The organic phase functions as a reservoir for toxic substrate styrene and intermediate styrene oxide. The time course on the production of (*R*)-MA from styrene is shown in Fig. 2b: as time went on, styrene concentration rapidly decreased and the concentration of (*R*)-MA and (*R*)-PED increased gradually. 1.07 g/L of (*R*)-MA was successfully obtained within the first 10 h, which corresponds to a space–time yield of 107 mg/L/h. After 24 h reaction, 1.52 g/L (10 mM) of (*R*)-MA was produced from 2.08 g/L (20 mM) of styrene, together with the remaining (*R*)-PED (800 mg/L; 6 mM) and styrene (300 mg/L; 3 mM). A small amount of mass imbalance (about 1 mM) was observed, which is probably due to styrene evaporation.

### Development of artificial enzyme cascade for the conversion of *L*-phenylalanine to (*R*)-mandelic acid

To develop an enzyme cascade for producing (*R*)-MA from *L*-Phe, deamination reaction catalyzed by phenylalanine ammonia lyase (PAL) from *Arabidopsis thaliana* and decarboxylation reaction catalyzed by phenylacrylic acid decarboxylase [PAD, consisting of ferulic acid decarboxylase (FDC1) and phenylacrylic acid decarboxylase (PAD1)] from *Aspergillus niger* were introduced into above mentioned epoxidation-hydrolysis-double oxidation artificial enzyme cascade, yielding a new five-enzyme cascade.

To engineer *E. coli* strains expressing the five-enzyme artificial cascade, the previously constructed pET-PAL-PAD (Zhou et al. 2016) were transformed into the *E. coli* (S2A) cells to give *E. coli* (P2S2A). The enzyme expression in *E. coli* (P2S2A) was analysed by SDS-PAGE (Additional file 1: Figure S19). The corresponding protein bands of PAL, PAD, SMO, StEH, and AldO were clearly observed, indicating these enzymes were well-expressed.

### Bioproduction of (*R*)-mandelic acid from *L*-phenylalanine via whole cell biotransformation in n-hexadecane-aqueous two-phase system

Cascade biotransformation of *L*-Phe to (*R*)-MA was conducted at 30 °C and 250 rpm using *E. coli* (P2S2A) cells (15 g cdw/L) in a mixture of KP buffer (200 mM, pH 8.0) containing 0.5% glucose and 2.48 g/L (15 mM) *L*-Phe and *n-*hexadecane (1:1; v/v). The organic phase functions as a reservoir for toxic intermediates such as styrene and styrene oxide. The time course of reaction is shown in Fig. 2c. With the rapid reduction of *L*-Phe, styrene, and (*R*)-PED and (*R*)-MA gradually increased. After six hours of the biotransformation, 650 mg/L of (*R*)-MA was produced, giving a space–time yield of 108.3 mg/L/h. 913 mg/L (6 mM) of (*R*)-MA was obtained after 24 h biotransformation of 2.48 g/L (15 mM) *L*-Phe. These results indicated the successful production of (*R*)-MA from *L*-Phe by the newly developed five-enzyme cascades. Considering the remaining *L*-Phe (3 mM), styrene (1 mM), and (*R*)-PED (4 mM), a small amount of mass imbalance (about 1 mM) was observed, which is also possibly due to styrene evaporation. The (*R*)-MA titer is much higher than those previously reported (236 mg/L in yeast cells and 680 mg/L in *E. coli* cells) (Reifenrath and Boles 2018; Sun et al. 2011). The biotransformation was also performed using 5 mM or 10 mM *L*-Phe under the same reaction condition, 3 mM and 6 mM of (*R*)-MA was produced with 60% conversion in both cases, which is higher than the conversion obtained from 15 mM *L*-Phe. Although the product titer is much higher than the previously reported, it is yet insufficient for commercial application. The expression of AldO was in a relatively low level when many different enzymes were co-expressed in *E. coli* cells (Fig. 1d). In addition, AldO showed the insufficient activity. These were probably the reasons for the accumulation of (*R*)-PED. Further optimizations, such as the expression of different enzymes in high level and at the good ratio, as well as engineering of much more efficient enzyme, are needed to increase the conversion, enhance the product titer, and reduce the accumulation of intermediates.

### Engineering of *E. coli* strains for the production of (*R*)*-*mandelic acid from glycerol or glucose

Direct fermentation of glucose or glycerol provides a prosperous approach for the green and sustainable biosynthesis of pharmaceuticals and high-value chemicals. For synthesis of (*R*)-MA from glycerol or glucose, the *E. coli* T7 host was changed into *E. coli* NST74 strain. The latter is a *L*-phenylalanine-overproducing strain, producing *L*-Phe from glycerol or glucose (Choi and Tribe 1982). The pRSF-AldO, pET-PAL-PAD, and pCDF-SMO-StEH were transformed into the previously engineered *E. coli* NST74(DE3) strain (Zhou et al. 2016) to give the *E. coli* NST74 (P2S2A) strain (Fig. 1c). The protein expression in *E. coli* NST74 (P2S2A) was analyzed by SDS-PAGE (Fig. 1d). Similar to the results from *E. coli* (P2S2A), all enzymes involved in the artificial enzyme cascade were well-expressed in *E. coli* NST74 (P2S2A) strain.

### Fermentative production of (*R*)*-*mandelic acid from glycerol or glucose using a* L*-phenylalanine-overproducing *E. coli* strain expressing the five-enzyme artificial cascade

**Fig. 3:**
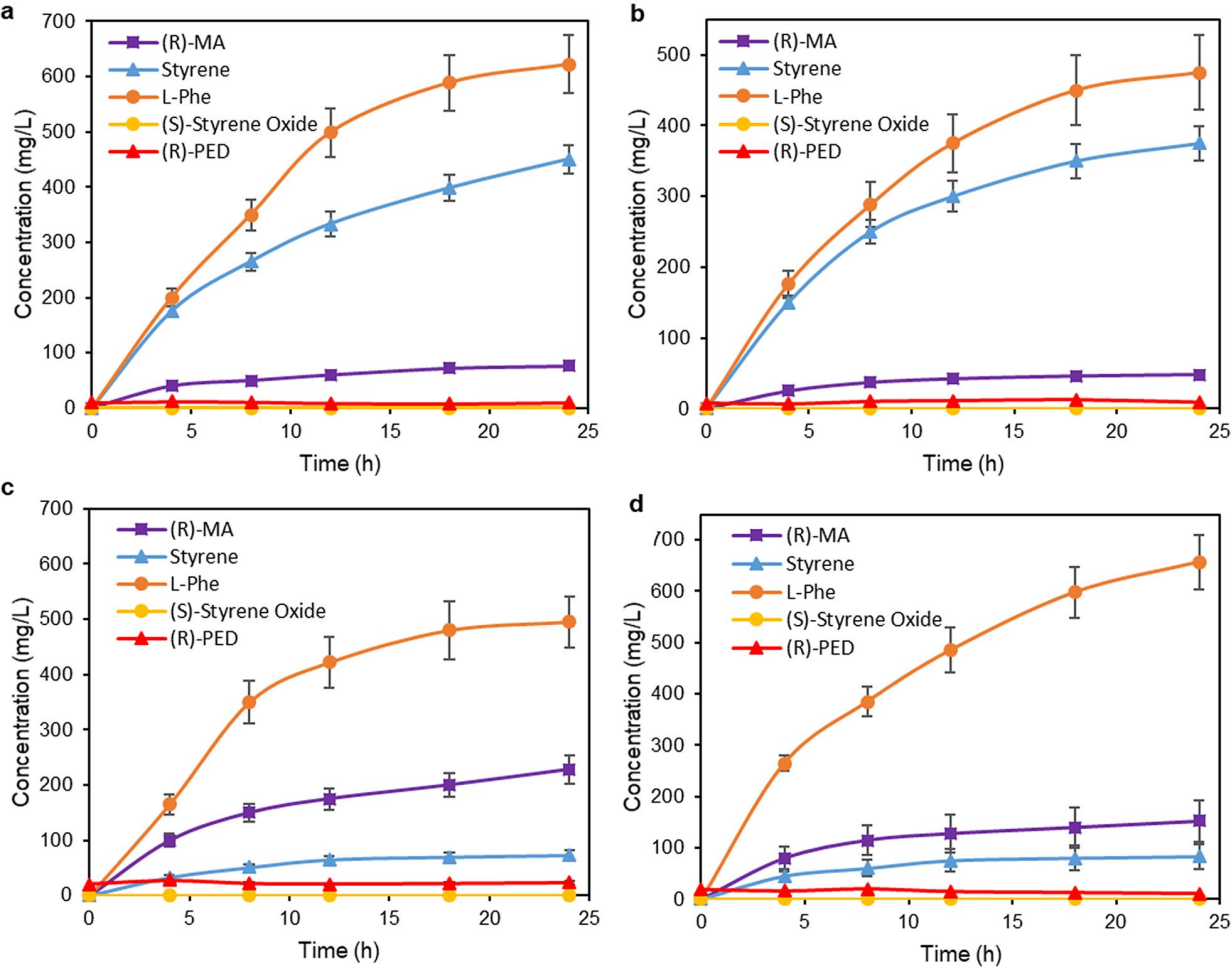
Time course of producing (*R*)-MA from glycerol or glucose with *E. coli* NST74 (P2S2A) at 25 °C. **a** Fermentation from glycerol in a single aqueous phase system. **b** Fermentation from glucose in a single aqueous phase system. **c** Fermentation from glycerol in *n-*hexadecane-aqueous phase system (1:2; v/v). **d** Fermentation from glucose in *n-*hexadecane-aqueous phase system (1:2; v/v)

Fermentative production of (*R*)*-*MA from glycerol or glucose in single *E. coli* strain was first investigated in aqueous system. *E. coli* NST74 (P2S2A) strain was grown in M9 (with glucose as the carbon source) or TB medium (with glycerol as the carbon source) containing respective antibiotics (50 µg/mL kanamycin, 50 µg/mL streptomycin, and 100 µg/mL ampicillin) at 37 °C and 250 rpm. When OD600_nm_ reached to ~ 0.6, 0.1 mM IPTG was added and then the cells were continued to grow at 25 °C for another 24 h. Samples were taken at different time points for monitoring the concentration of *L*-Phe, styrene and (*R*)-MA in medium. Fermentative biotransformation of glycerol (Fig. 3a) and glucose (Fig. 3b) successfully gave (*R*)-MA concentration of 75 and 48 mg/L, respectively. The fermentation of glycerol gave higher (*R*)-MA titer in comparison with the one performed with glucose as carbon source, suggesting that the cells tend to consume the glucose more for their growth than for the (*R*)-MA production. Notably, high accumulation of styrene in the aqueous phase was observed during the fermentation, which caused the toxicity to the cells.

To tackle the styrene toxicity issue, *n-*hexadecane-aqueous two-phase system was used for the fermentation. *E. coli* NST74 (P2S2A) cells were grown in M9 or TB medium under the above-mentioned conditions. After the addition of IPTG, *n-*hexadecane was added into the aqueous medium at a ratio of 1:2. The reaction was then conducted at 25 °C and 250 rpm for 24 h. Time courses of the fermentation are shown in Fig. 3c, d. The concentration of (*R*)-PED and (*S*)-styrene oxide were very low, as shown in Fig. 3, possibly caused by the low concentration of *L*-Phe produced from glycerol or glucose. Compared to previous single aqueous system; however, the two-phase system still significantly increased the product concentration. While the *L*-phenylalanine, (*R*)-PED, and (*R*)-MA were formed in the aqueous phase, the intermediates styrene and (*S*)-styrene oxide remained in the organic phase to avoid the toxicity issue. As the best results, 228 and 152 mg/L of (*R*)-MA was obtained from the fermentative biotransformation of glycerol and glucose in two-phase system, respectively, with the significant decrement of styrene accumulation.

### Production of (*R*)-mandelic acid from glycerol or glucose by coupling of two *E. coli* strains expressing the artificial enzyme cascade and *L-*phenylalanine biosynthesis pathway, respectively

**Fig. 4:**
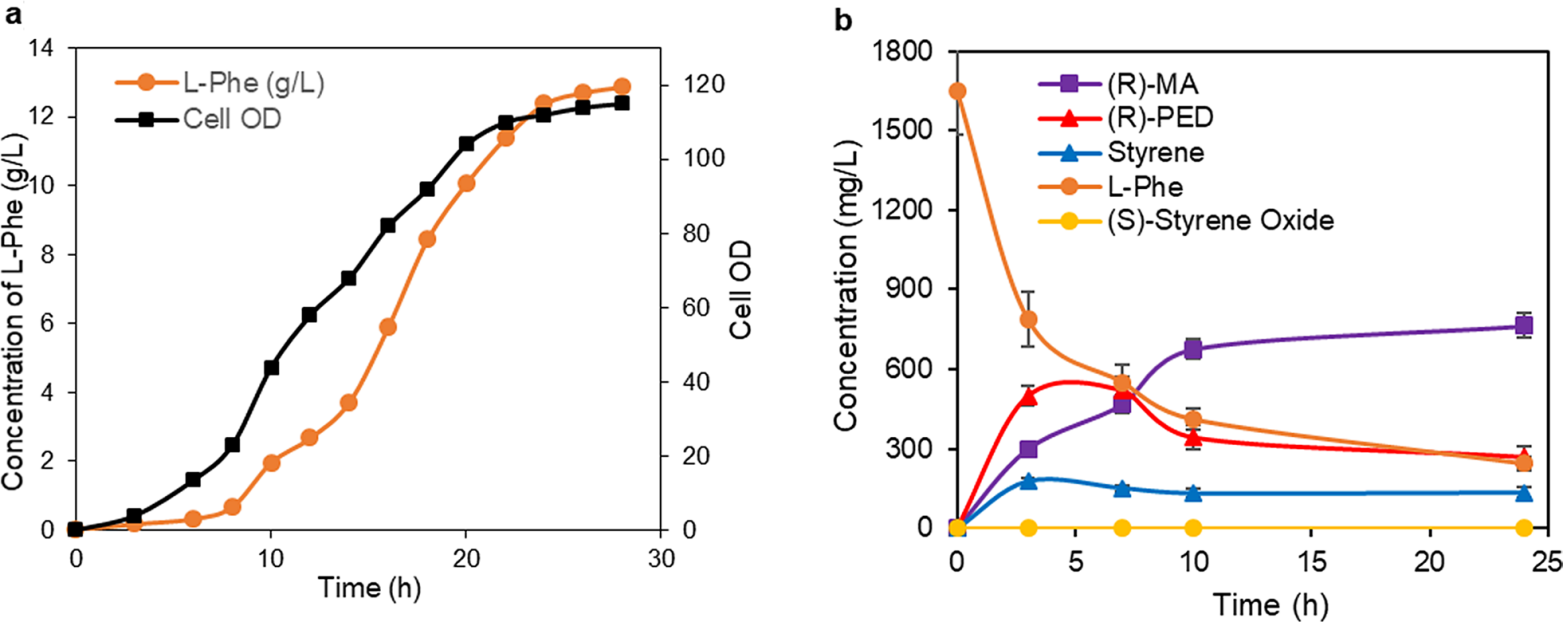
Time course of producing (*R*)-MA from glycerol via coupled *E. coli* (NST74-Phe) and *E. coli* (P2S2A). **a** Fermentative production of *L*-Phe from glycerol with *E. coli* (NST74-Phe). **b** Biotransformation of *L-*Phe to (*R*)-MA by adding *E. coli* (P2S2A) cells into glycerol-fermented media of *E. coli* (NST74-Phe)

Production of (*R*)-MA from glycerol or glucose was examined by coupling *E. coli* NST74-Phe, which over-expressed five limiting enzymes to convert glycerol or glucose to *L-*Phe via Shikimate pathway (Sekar et al. 2019), and *E. coli* (P2S2A) expressing the novel artificial cascade to convert *L*-Phe to (*R*)-MA (Fig. 2c). *E. coli* NST74-Phe was first grown in modified NH_4_-media containing 10 g/L glycerol or glucose for 28 h to produce 12.87 g/L (Fig. 4a) and 11 g/L of *L-*Phe from glycerol and glucose, respectively. Freshly made *E. coli* (P2S2A) cells, concentrated KP buffer (pH 8.0), and glucose solution were directly added into fermentation medium, resulting in a reaction mixture containing 1.65 g/L of *L-*Phe, 200 mM KP buffer (pH 8.0), 1% glucose, and 15 g cdw/L of *E. coli* (P2S2A) cells. *n*-Hexadecane was added to give a ratio of 1:1 (v/v) for aqueous and organic phases. The biotransformation was performed at 30 °C and 250 rpm for 24 h. Nevertheless, 760 and 455 mg/L of (*R*)-MA was obtained from glycerol-fermented media and glucose-fermented media, respectively (Fig. 4b and Additional file 1: Fig. S18). While the optimal temperature for expression and production of *L*-phe from glycerol or glucose is 37 °C (Sekar et al. 2019), the optimal temperature for the expression of artificial enzyme cascade is 22 °C and for cascade biotransformation of *L*-Phe to (*R*)-MA is 30 °C. Therefore, the single-cell fermentation at 25 °C gave lower product concentration. In contrary, the two-cells system could be operated at their respective optimum temperatures to give higher productivity. The results with the use of glycerol as feedstock showed higher (*R*)-MA titer than those previously reported (236 mg/L in yeast cells and 680 mg/L in *E. coli* cells) (Reifenrath and Boles 2018; Sun et al. 2011).

### Preparative Biotransformation of *L-*Phe to (*R*)-MA in aqueous-*n-*hexadecane two-phase system

Preparative biotransformation of *L*-Phe to (*R*)-MA was performed in two-phase system with *E. coli* (P2S2A) cells, yielding 6 mM (*R*)-MA in 24 h with 60% conversion. After purification, pure product was obtained with an isolated yield of 46%. ^1^H and ^13^C NMR analysis confirmed the chemical structure of (*R*)-MA products (Additional file 1: Figure S17). Chiral HPLC analysis showed > 99% *ee* of the produced (*R*)-MA (Additional file 1: Figure S16).

## Conclusions

A novel, green, and efficient three-enzyme artificial enzyme cascade was developed to produce (*R*)-mandelic acid at 1.52 g/L from styrene in > 99% *ee*. By additionally introducing deamination and decarboxylation reactions into the above cascade, a new five-enzyme artificial cascade was developed for sustainable production of (*R*)-MA from *L-*Phe. Engineered *E. coli* strain expressing the five-enzyme cascade successfully gave 913 mg/L (*R*)-MA with > 99% *ee*, representing the highest (*R*)-MA concentration among bioproduction from natural resources. Combining artificial enzyme cascade and *L-*Phe biosynthesis pathways in a single *E. coli* strain led to one-pot synthesis of (*R*)-MA from glycerol or glucose at 228 and 152 mg/L, respectively. Enhanced (*R*)-MA production (760 mg/L from glycerol and 455 mg/L from glucose) was achieved by coupling two *E. coli* strains expressing *L*-Phe-producing pathway and the artificial cascade.

### Supplementary Information

**Additional file 1**: **Table S1**. Strains used in this study. **Table S2**. Enzymes used in this study. **Fig. S1**. Standard calibration curve for GC analysis of styrene. **Fig. S2**. Standard calibration curve for GC analysis of (*S*)-styrene oxide. **Fig. S3**. GC chromatogram of styrene (standard) with benzyl alcohol as internal standard (I.S.). **Fig. S4**. GC chromatogram of (*S*)-styrene oxide (standard) with benzyl alcohol as internal standard (I.S.). **Fig. S5**. Standard calibration curve for HPLC analysis of *L*-Phe. **Fig. S6**. Standard calibration curve for HPLC analysis of (*R*)-PED. **Fig. S7**. Standard calibration curve for HPLC analysis of (*R*)-MA. **Fig. S8**. HPLC chromatogram of *L*-Phe (standard) with benzyl alcohol as internal standard (I.S.). **Fig. S9**. HPLC chromatogram of (*R*)-PED (standard) with benzyl alcohol as internal standard (I.S.). **Fig. S10**. HPLC chromatogram of (*R*)-MA (standard) with benzyl alcohol as internal standard (I.S.). **Fig. S11**. HPLC chromatogram of (*R*)-MA produced during the biotransformation of (*R*)-PED (20 mM) with resting cells of *E. coli* (AldO) cells (15 g cdw/L) in KP buffer (200 mM, pH 8.0) at 30 °C for 24 h. Benzyl alcohol was used as internal standard (I.S.). **Fig. S12**. HPLC chromatogram of (*R*)-MA produced during the biotransformation of styrene (20 mM) with resting cells of *E. coli* (S2A) cells (15 g cdw/L) in a mixture of KP buffer (200 mM, pH 8.0) containing 0.5% glucose and n-hexadecane (1:1, v/v) at 30 °C for 24 h. Benzyl alcohol was used as internal standard (I.S.). **Fig. S13**. HPLC chromatogram of (*R*)-MA produced during the biotransformation of *L*-Phe (15 mM) with resting cells of *E. coli* (P2S2A) cells (15 g cdw/L) in a mixture of KP buffer (200 mM, pH 8.0) containing 0.5% glucose and n-hexadecane (1:1, v/v) at 30 °C for 24 h. Benzyl alcohol was used as internal standard (I.S.). **Fig. S14**. HPLC chromatogram of (*R*)-MA formed after the biotransformation from glycerol with growing cells of *E. coli* NST74 (P2S2A). Reaction was performed in a mixture of KP buffer (200 mM, pH 8.0) and n-hexadecane (2:1, v/v) at 25 °C for 24 h. Benzyl alcohol was used as internal standard (I.S.). **Fig. S15**. HPLC chromatogram of (*R*)-MA produced from glycerol via coupling *E. coli* NST74-Phe cells for the production of *L*-Phe and *E. coli* (P2S2A) cells (15 g cdw/L) for the production of (*R*)-MA in a reaction mixture containing KP buffer (200 mM, pH 8.0, 0.5% glucose) and n-hexadecane (1:1, v/v) at 30 °C for 24 h. Benzyl alcohol was used as internal standard (I.S.). **Fig. S16**. Chiral HPLC chromatograms of (*R*)-MA. a) Racemic MA standard, b) (*R*)-MA standard, c) Isolated (*R*)-MA products from biotransformation of *L*-Phe to (*R*)-MA with *E. coli* (P2S2A) cells. **Fig S17**. NMR spectrum of (*R*)-MA prepared from biotransformation of *L*-Phe with *E. coli* (P2S2A) cells. a) ^1^H NMR spectrum (400 MHz, CD_3_OD), δ 7.47 (dd, J = 15.1, 7.2 Hz, 2H), 7.31 (ddd, J = 10.7, 9.0, 5.1 Hz, 3H), 5.19 (s, 1H). b) ^13^C NMR spectrum (101 MHz, CD_3_OD), δ 176.27, 140.75, 129.53, 129.33, 127.96, 74.21. **Fig. S18**. Time course of biotransformation of glucose to (*R*)-MA via coupling *E. coli* NST74-Phe and *E. coli* (P2S2A) cells in aqueous-n-hexadecane two-phase system. **Fig. S19**. SDS-PAGE analysis of total intracellular protein of *E. coli* cells used for the bioproduction of (*R*)-MA; Lane A: *E. coli* (AldO); B: *E. coli* (S2A); C: *E. coli* (P2S2A).

## Data Availability

The data and the materials are all available in this article as well as the supporting information.

## Abbreviations

*L-*Phe: *L*-Phenylalanine
MA: Mandelic acid
PGA: Phenylglyoxylic acid
NLases: Nitrilases
AldO: Alditol oxidase
SMO: Styrene monooxygenase
StEH: Epoxide hydrolase
PAL: Phenylalanine ammonia lyase
PAD: Phenylacrylic acid decarboxylase
PED: 1,2-Phenylethanediol
*ee*: Enantiomeric excess
IPTG: Isopropyl β-D-1-thiogalactopyranoside
PCR: Polymerase Chain Reaction
KP buffer: Potassium phosphate buffer
HPLC: High-Performance Liquid Chromatography
SDS-PAGE: Sodium dodecyl sulfate polyacrylamide gel electrophoresis
