# Supplementary material for: Production of (R)-mandelic acid from styrene, L-phenylalanine, glycerol, or glucose via cascade biotransformations
Source: Bioresour Bioprocess. 2021 Mar 4;8(1):22. doi: 10.1186/s40643-021-00374-6 (PMC10992357; doi:10.1186/s40643-021-00374-6)
Supplement: Supplementary file 1 — Additional file 1: Table S1. Strains used in this study. Table S2. Enzymes used in this study. Fig. S1. Standard calibration curve for GC analysis of styrene. Fig. S2. Standard calibration curve for GC analysis of (S)-styrene oxide. Fig. S3. GC chromatogram of styrene (standard) with benzyl alcohol as internal standard (I.S.). Fig. S4. GC chromatogram of (S)-styrene oxide (standard) with benzyl alcohol as internal standard (I.S.). Fig. S5. Standard calibration curve for HPLC analysis of L-Phe. Fig. S6. Standard calibration curve for HPLC analysis of (R)-PED. Fig. S7. Standard calibration curve for HPLC analysis of (R)-MA. Fig. S8. HPLC chromatogram of L-Phe (standard) with benzyl alcohol as internal standard (I.S.). Fig. S9. HPLC chromatogram of (R)-PED (standard) with benzyl alcohol as internal standard (I.S.). Fig. S10. HPLC chromatogram of (R)-MA (standard) with benzyl alcohol as internal standard (I.S.). Fig. S11. HPLC chromatogram of (R)-MA produced during the biotransformation of (R)-PED (20 mM) with resting cells of E. coli (AldO) cells (15 g cdw/L) in KP buffer (200 mM, pH 8.0) at 30 °C for 24 h. Benzyl alcohol was used as internal standard (I.S.). Fig. S12. HPLC chromatogram of (R)-MA produced during the biotransformation of styrene (20 mM) with resting cells of E. coli (S2A) cells (15 g cdw/L) in a mixture of KP buffer (200 mM, pH 8.0) containing 0.5% glucose and n-hexadecane (1:1, v/v) at 30 °C for 24 h. Benzyl alcohol was used as internal standard (I.S.). Fig. S13. HPLC chromatogram of (R)-MA produced during the biotransformation of L-Phe (15 mM) with resting cells of E. coli (P2S2A) cells (15 g cdw/L) in a mixture of KP buffer (200 mM, pH 8.0) containing 0.5% glucose and n-hexadecane (1:1, v/v) at 30 °C for 24 h. Benzyl alcohol was used as internal standard (I.S.). Fig. S14. HPLC chromatogram of (R)-MA formed after the biotransformation from glycerol with growing cells of E. coli NST74 (P2S2A). Reaction was performed in a mixture of KP buffer (200 mM, p [file 40643_2021_374_MOESM1_ESM.docx]

Supporting Information

**Production of (*R*)-Mandelic Acid from Styrene, *L-*Phenylalanine, Glycerol, or Glucose *via* Cascade Biotransformations**

Benedict Ryan Lukito^1^, Zilong Wang^1,2^, Balaji Sundara Sekar^1,2^, Zhi Li^1,2^*

^1^ Department of Chemical and Biomolecular Engineering, National University of Singapore, Singapore 117585, Singapore

^2^ Synthetic Biology for Clinical and Technological Innovation (SynCTI), Life Sciences Institute, National University of Singapore, Singapore 117456, Singapore

* Corresponding author.

E-mail address: [chelz@nus.edu.sg](mailto:chelz@nus.edu.sg) (Z. L.)

**Table of Contents**

[1. Chemicals and media 3](#_Toc62296424)

[2. Analytical methods 3](#_Toc62296425)

[3. Strains and enzymes used in this study 4](#_Toc62296426)

[4. General procedure of fed-batch growing cells fermentation for the production of *L-*Phe from glycerol or glucose 5](#_Toc62296427)

[5. General procedure for culturing *E. coli* cells 6](#_Toc62296428)

[6. Preparative biotransformation of *L*-Phe to (*R*)-MA in aqueous-organic two-phase system 6](#_Toc62296429)

[7. Supporting Figures 7](#_Toc62296430)

[8. References 17](#_Toc62296431)

# Chemicals and media

*D*-glucose (99%), glycerol (98%), *L-*phenylalanine (99%), styrene (99%), (*R*)-1,2-phenylethanediol (98%), (*R*)-mandelic acid (99%), (*S*)-styrene oxide (97%), n-hexadecane (99%), streptomycin sulfate salt (95%), kanamycin, chloramphenicol, ampicillin sodium salt (96%), trifluoro acetic acid (99%), MgSO_4_ (99%), FeCl_3_ (99%), NaCl (99.5%), KH_2_PO_4_ (99%), benzyl alcohol (99%), Na_2_HPO_4_•2H_2_O (99%), CaCl_2_ (99%), NH_4_Cl (99%), NaOH (99%), Na_2_SO_4_ (99%), NaMoO_4_ (99%), CoCl_2_•6H_2_O (99%), HCl (99%), H_3_BO_4_ (99%), CuCl_2_ (99%), and ZnCl_2_•4H_2_O (98%) were purchased from Sigma Aldrich. Isopropyl β-D-1-thiogalactopyranoside (99%) was bought from Gold Biotechnology. K_2_HPO_4_•3H_2_O (99%) was bought from Merck. Acetonitrile (HPLC grade) and ethyl acetate (HPLC grade) were purchased from Tedia.

Luria broth medium and yeast extract were purchased from Biomed Diagnostics. M9 was used as a culture medium, containing (per Liter): 6 g yeast extract, 20 g glucose, 0.5 g NaCl, 6 g Na_2_HPO_4_, 0.1 mM CaCl_2_, 1 mM MgSO_4_, 1 mL trace metals, 1 g NH_4_Cl, and 3 g KH_2_PO_4_. Terrific broth (TB) medium was used as alternative culture medium, containing (per Liter): 24 g yeast extract, 20 g tryptone, 4 mL glycerol.

# Analytical methods

*L-*phenylalanine (*L*-Phe), (*R*)-1,2-phenylethanediol (PED), and (*R*)-mandelic acid (MA) were analyzed using reverse phase HPLC (Shimadzu Prominence HPLC System). Column: Agilent Poroshell 120 SB-C18 (150 mm x 4.6 mm x 2.7 µm). Mobile phase: 30% acetonitrile (ACN) and 70% water containing 0.1% trifluoro acetic acid (TFA). Flow rate: 0.4 ml/min, temperature: 25^o^C. Detector: photodiode array detector. Wavelength: 210 nm. Retention time: 5.5 min for (*R*)-1,2-phenylethanediol, 5.8 min for (*R*)-mandelic acid, 4.1 min for *L-*phenylalanine, and 8.3 min for benzyl alcohol (internal standard).

Styrene and (*S*)*-*styrene oxide were analyzed using Agilent 7890A Gas Chromatography (GC). Column: Agilent HP-5 (30 m x 0.32 mm x 0.25 mm). Temperature programme: initial temperature at 70^o^C, increase 25^o^C/min until it reached 200^o^C; subsequently increase to 250^o^C at 50^o^C/min, then hold for 1 minute; Lastly, increase to 270^o^C at 20^o^C/min. Retention time: 2.8 min for styrene, 4.0 min for (*S*)­-styrene oxide, and 3.8 min for benzyl alcohol (internal standard).

The *ee* of (*R*)-MA was determined by normal phase HPLC with a chiral column. Column: Daicel Chiralpak IC-3 (250 × 4.6 mm, 3 μm). Mobile phase: 10% isopropyl alcohol and 90% *n*-hexane. Flow rate: 0.8 mL/min. Detection: 210 nm. Temperature: 25 °C.

# Strains and enzymes used in this study

**Table S1**. Strains used in this study

| **Strains** | **Genotpye and plasmids contained** | **Source** |
| --- | --- | --- |
| *E. coli* T7 | *fhuA2 lacZ::T7 gene1 [lon] ompT gal sulA11 R(mcr-73::miniTn10--TetS)2 [dcm] R(zgb-210::Tn10--TetS) endA1 Δ(mcrC-mrr)114::IS10* | New England Biolabs |
| *E. coli* NST74 | *aroH367, tyrR366, tna-2, lacY5, aroF394(fbr), malT384, pheA101(fbr) ,pheO352, aroG397(fbr)* | ATCC 31884 |
| *E. coli* NST74 (DE3) | *aroH367, tyrR366, tna-2, lacY5, aroF394(fbr), malT384, pheA101(fbr) ,pheO352, aroG397(fbr) (DE3)* | (Zhou et al. 2018) |
| *E. coli* (AldO) | pRSF-AldO | This study |
| *E. coli* (S2A) | pCDF-SMO-StEH, pRSF-AldO | This study |
| *E. coli* (P2S2A) | pET-PAL-PAD, pCDF-SMO-StEH, pRSF-AldO | This study |
| *E. coli* NST74 (P2S2A) | pET-PAL-PAD, pCDF-SMO-StEH, pRSF-AldO | This study |
| *E. coli* NST74-Phe | pCDF-Phe | (Sekar et al. 2019) |

**Table S2**. Enzymes used in this study

| **Enzyme** | **Reaction** | **Source** | **Descriptions** | **References** |
| --- | --- | --- | --- | --- |
| Phenylalanine ammonia lyase (PAL) | Convert *L*-Phe to *trans-*cinnamic acid | *Arabidopsis thaliana* | Encoded by *Atpal* 2 | (Cochrane et al. 2004) |
| Phenylacrylic acid decarboxylase (PAD) | Convert *trans-*cinnamic acid to styrene | *Aspergillus niger* | PAD consists of ferulic acid decarboxylase (FDC1) and phenylacrylic acid decarboxylase (PAD1), which are encoded by *fdc1* and *pad1*, respectively | (Payne et al. 2015) |
| Styrene monooxygenase (SMO) | Convert styrene to (*S*)-styrene oxide | *Pseudomonas sp. VLB120* | SMO consists of StyA and StyB, which are encoded by *styA* and *styB*, respectively | (Panke et al. 1998) |
| Epoxide hydrolase (StEH) | Convert (*S*)-styrene oxide to (*R*)-PED | *Solanum tuberosum* | Encoded by *Steh* 1 | (Lindberg et al. 2008) |
| Alditol oxidase (AldO) | Convert (*R*)-PED to (*R*)-MA | *Streptomyces coelicolor* A3(2) | The gene of AldO was codon optimized and synthesized based on the reported gene sequence (GenBank accession No. SCO6147) | (Dominic P. H. M. Heuts 2007; van Hellemond et al. 2009) |

# General procedure of fed-batch growing cells fermentation for the production of *L-*Phe from glycerol or glucose

*E. coli* NST74-Phe was inoculated in 100 mL LB medium containing 50 µg/mL streptomycin at 37^o^C and 250 rpm for 12 h. The inoculated strain was subsequently transferred and cultured in 1.5-liter bioreactor with the modified NH_4_-media containing 10 g/L (NH_4_)_2_SO_4_, 5 g/L KH_2_PO_4_, 5 g/L MgSO_4_, 5 g/L yeast extract, 50 µg/mL streptomycin, and 10 g/L carbon source (glycerol or glucose) for 28 h. 30% NH_4_OH and 80% carbon sources were used as feed to control the carbon source at pH 6.8 and 5-10 g/L, respectively, without acetate accumulation. Aeration and stirrer speed were also modified between 0.5-3 litre per minute and 500-2000 rpm, respectively, to maintain the dissolved oxygen (DO) level at 20-30%. IPTG was added to a final concentration of 0.1 mM at 8 h to induce the protein expression. In the end of the fermentation, 30% NH_4_OH was added to adjust the reaction mixture to pH 8.0 before performing further biotransformation.

# General procedure for culturing *E. coli* cells

*E. coli* strains were inoculated with LB medium containing appropriate antibiotics (50 µg/mL kanamycin, 50 µg/mL chloramphenicol, 50 µg/mL streptomycin, or 100 µg/mL ampicillin) at 37^o^C and 250 rpm for 8-10 hours. The inoculated strain was subsequently transferred and grown into a 250 mL-baffled culture flask with M9 medium containing 6 g/L yeast extract, 20 g/L glucose, and appropriate antibiotics in a total volume of 50 mL. IPTG was added at 2 h to a final concentration of 0.5 mM to induce the protein expression at 22^o^C. The cells were harvested after 14 h of growth by centrifugation (4000*g*, 10 min).

# Preparative biotransformation of *L*-Phe to (*R*)-MA in aqueous-organic two-phase system

50 mL KP buffer (100 mM, pH 8.0) containing *E. coli* (P2S2A) cells (15 g cdw/L), 0.5% glucose, and 10 mM *L-*Phe was mixed with 50 mL *n*-hexadecane to perform the biotransformation at 30^o^C and 250 rpm for 24 h. The reaction mixture was subsequently centrifuged (5000 *g,* 15 min) to separate the aqueous phase from the organic phase and the cell pellet. The aqueous phase was collected, saturated with NaCl, and adjusted to pH 1 by adding 10 M HCl, followed by the extraction with ethyl acetate (3x50 mL). The organic phase was then collected and dried using Na_2_SO_4_, followed by the filtration. The organic phase was subsequently evaporated by using the rotary evaporator to remove the ethyl acetate. The crude product was subjected to crystallization in ethyl acetate by dissolving at 65 °C and slowly cooling down to -20 °C. The crystals were taken by filtration, and the mother liquor was evaporated and subjected to crystallization again. The collected crystals were dried under vacuum for 12 h for further chiral HPLC and NMR analysis.

# Supporting Figures

**Fig. S1.** Standard calibration curve for GC analysis of styrene

**Fig. S2.** Standard calibration curve for GC analysis of (*S*)-styrene oxide

**
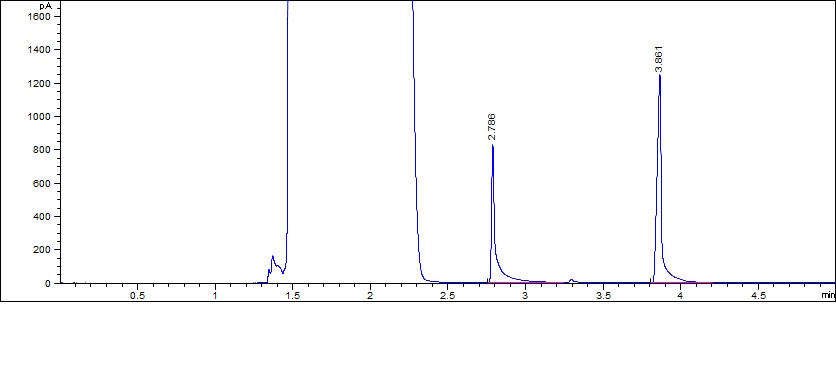
**

I.S

**Styrene**

**Fig. S3.** GC chromatogram of styrene (standard) with benzyl alcohol as internal standard (I.S.)

**
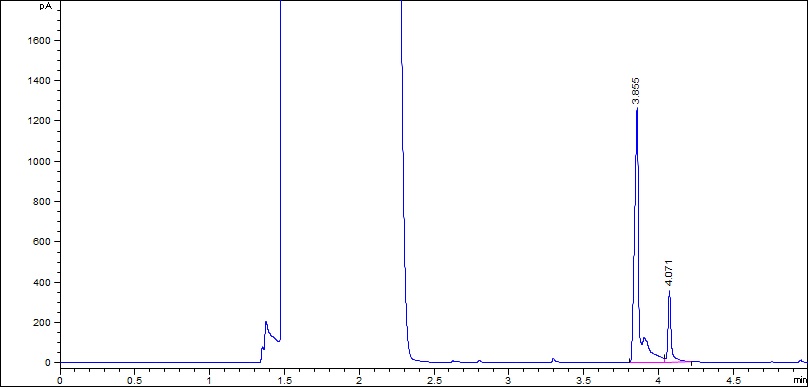
**

(*S*)-Styrene Oxide

I.S

**Fig. S4.** GC chromatogram of (*S*)*-*styrene oxide (standard) with benzyl alcohol as internal standard (I.S.)

**Fig. S5.** Standard calibration curve for HPLC analysis of *L*-Phe

**Fig. S6.** Standard calibration curve for HPLC analysis of (*R*)-PED

**Fig. S7.** Standard calibration curve for HPLC analysis of (*R*)*-*MA


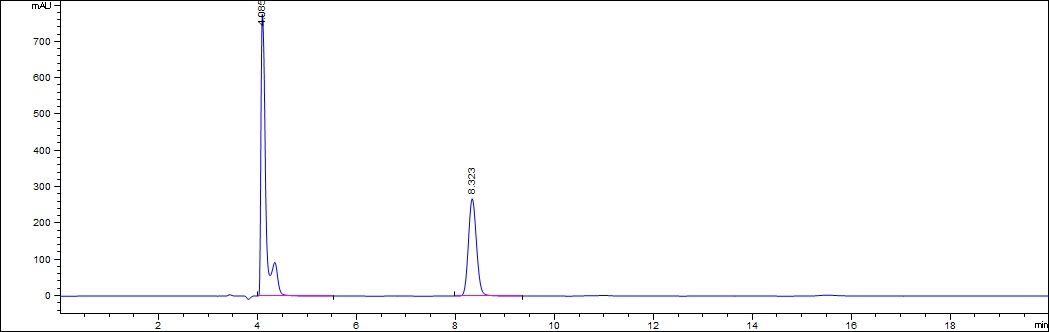


*L-*Phe

I.S.

**Fig. S8.** HPLC chromatogram of *L*-Phe (standard) with benzyl alcohol as internal standard (I.S.)

**
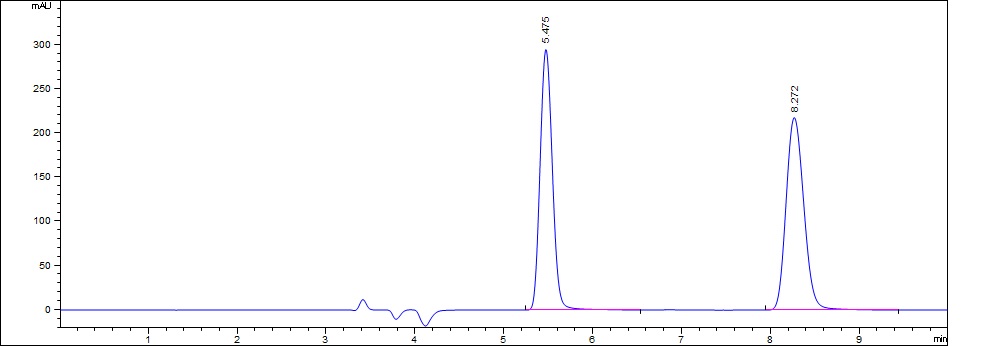
**

(*R*)*-*PED

I.S.

**Fig. S9.** HPLC chromatogram of (*R*)-PED (standard) with benzyl alcohol as internal standard (I.S.)


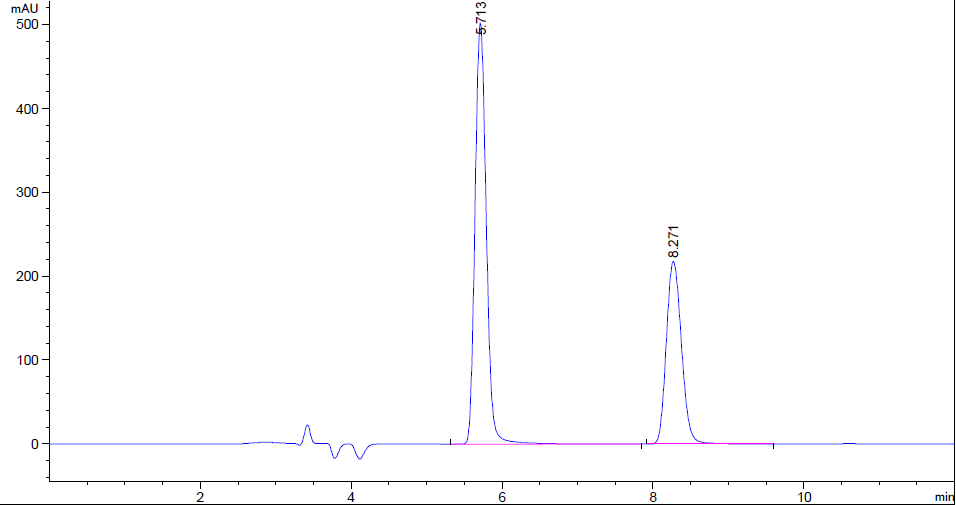


(*R*)*-*MA

I.S.

**Fig. S10.** HPLC chromatogram of (*R*)-MA (standard) with benzyl alcohol as internal standard (I.S.)


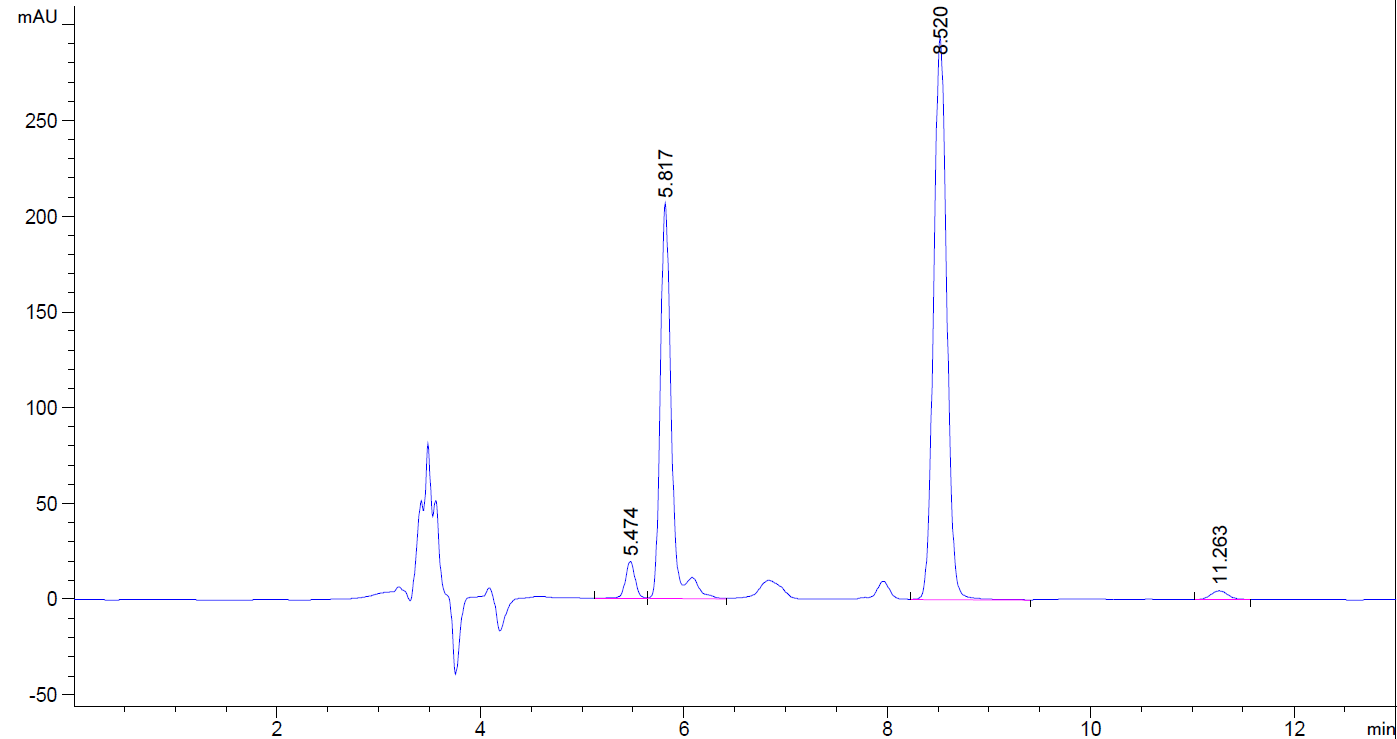


(*R*)*-*MA

(*R*)*-*PED

I.S.

**Fig. S11.** HPLC chromatogram of (*R*)*-*MA produced during the biotransformation of (*R*)-PED (20 mM) with resting cells of *E. coli* (AldO) cells (15 g cdw/L) in KP buffer (200 mM, pH 8.0) at 30^o^C for 24 h. Benzyl alcohol was used as internal standard (I.S.)


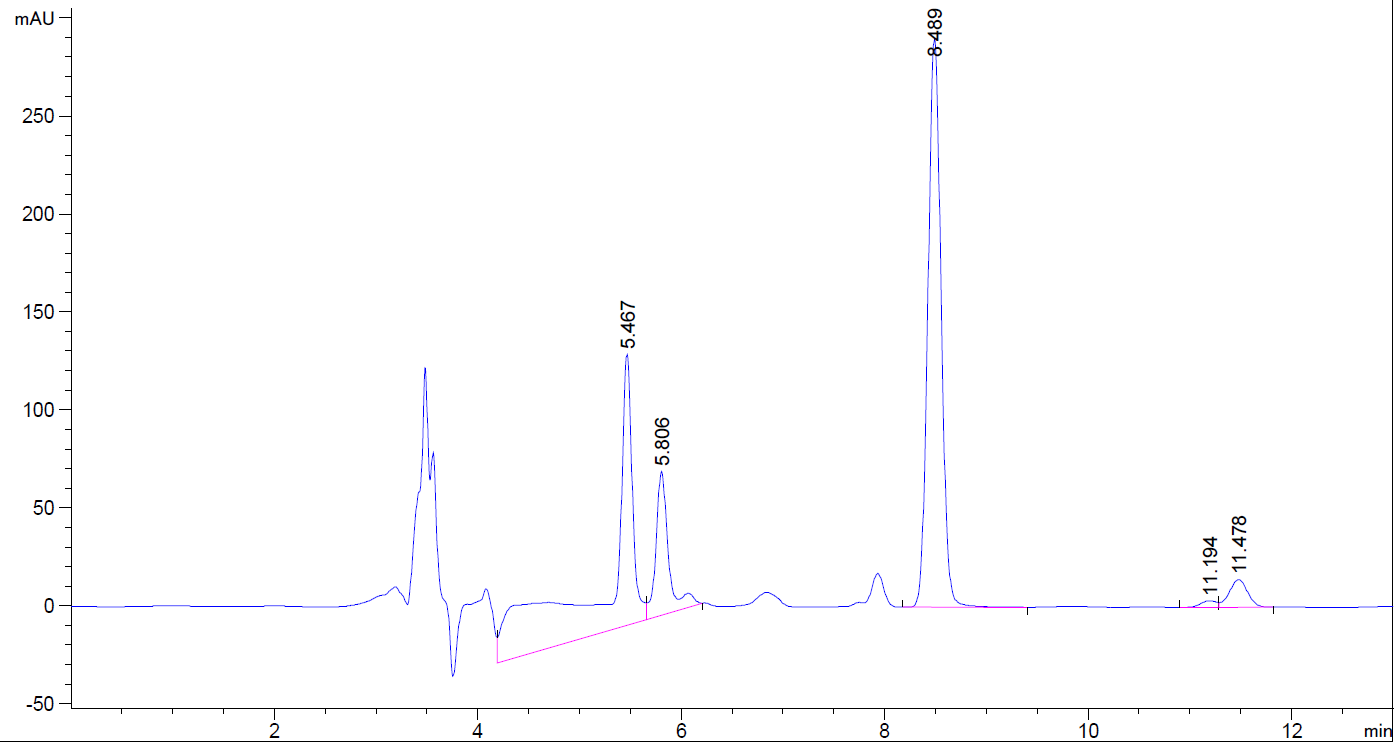


(*R*)*-*MA

I.S.

(*R*)*-*PED

**Fig. S12.** HPLC chromatogram of (*R*)*-*MA produced during the biotransformation of styrene (20 mM) with resting cells of *E. coli* (S2A) cells (15 g cdw/L) in a mixture of KP buffer (200 mM, pH 8.0) containing 0.5% glucose and *n*-hexadecane (1:1, v/v) at 30^o^C for 24 h. Benzyl alcohol was used as internal standard (I.S.)


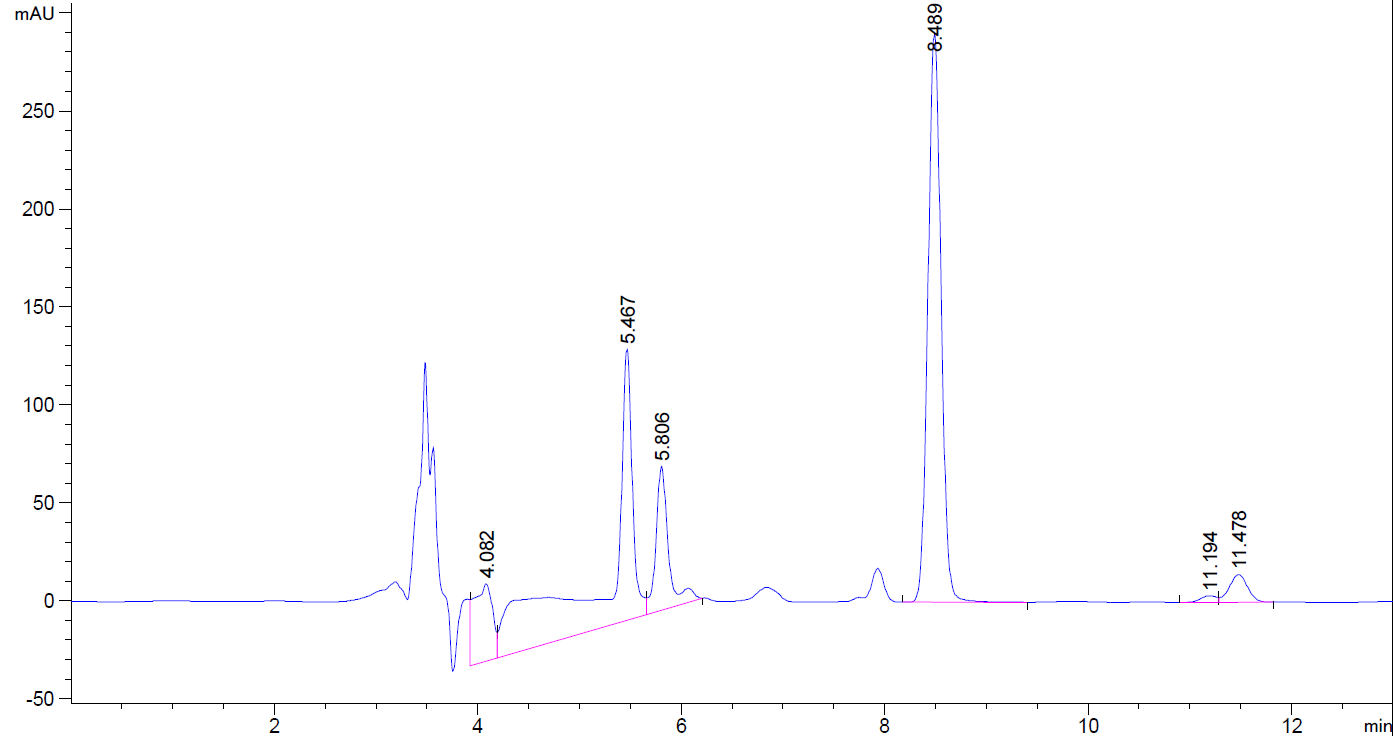


(*R*)*-*MA

I.S.

(*R*)*-*PED

**Fig. S13.** HPLC chromatogram of (*R*)*-*MA produced during the biotransformation of *L*-Phe (15 mM) with resting cells of *E. coli* (P2S2A) cells (15 g cdw/L) in a mixture of KP buffer (200 mM, pH 8.0) containing 0.5% glucose and *n*-hexadecane (1:1, v/v) at 30^o^C for 24 h. Benzyl alcohol was used as internal standard (I.S.)


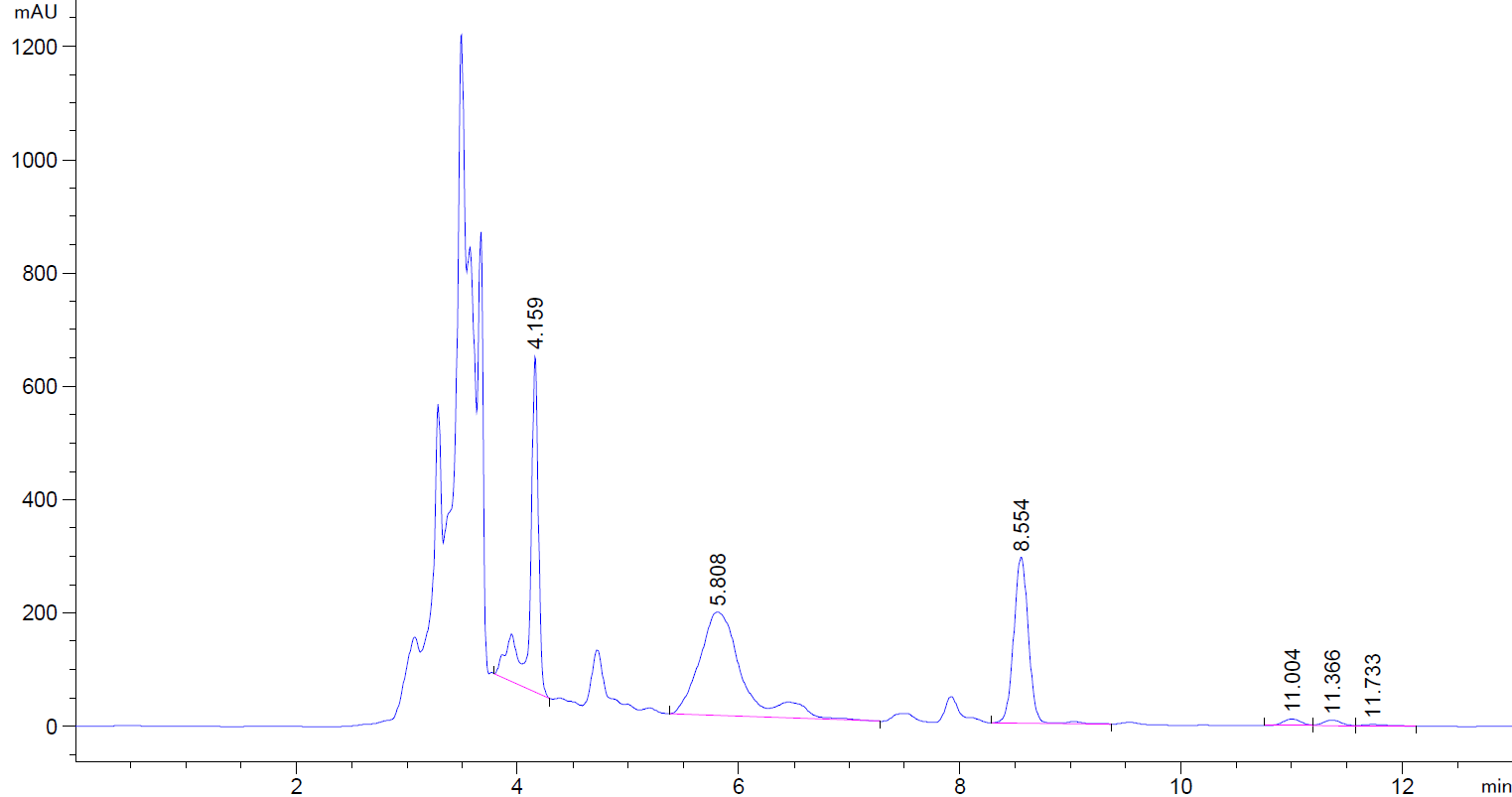


(*R*)*-*MA

I.S.

**Fig. S14.** HPLC chromatogram of (*R*)-MA formed after the biotransformation from glycerol with growing cells of *E. coli* NST74 (P2S2A). Reaction was performed in a mixture of KP buffer (200 mM, pH 8.0) and *n*-hexadecane (2:1, v/v) at 25^o^C for 24 h. Benzyl alcohol was used as internal standard (I.S.)


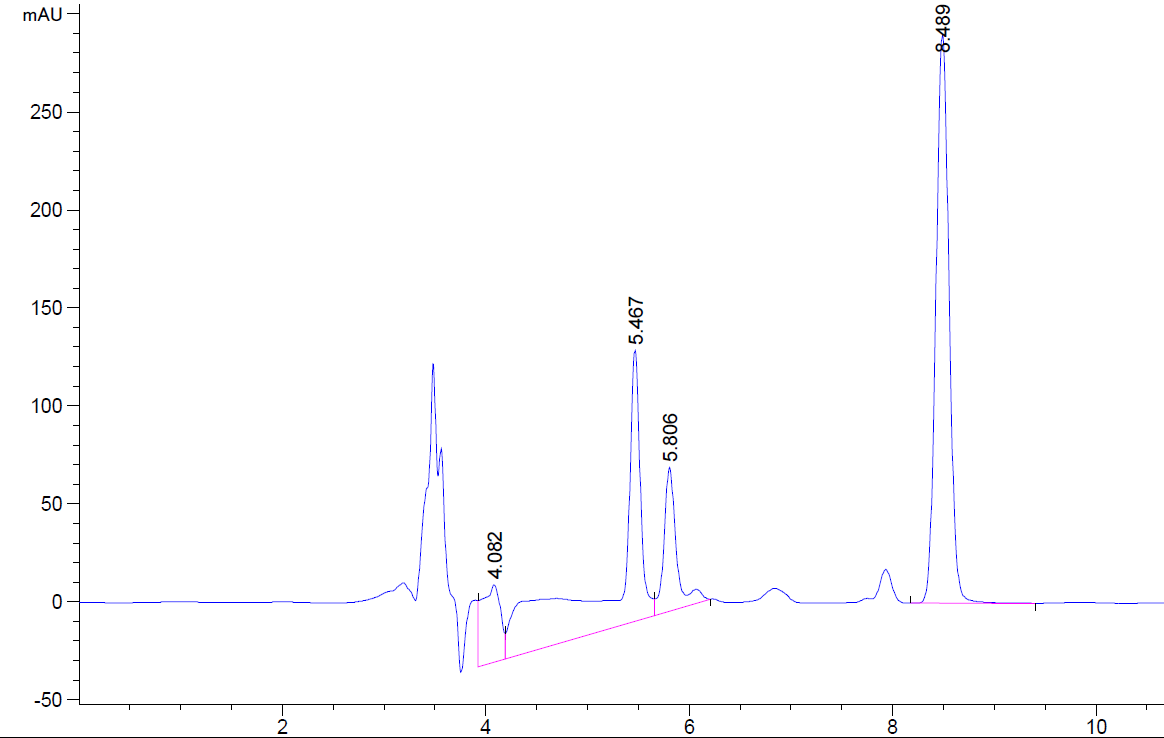


(*R*)*-*PED

I.S.

(*R*)*-*MA

**Fig. S15.** HPLC chromatogram of (*R*)-MA produced from glycerol *via* coupling *E. coli* NST74-Phe cells for the production of *L-*Phe and *E. coli* (P2S2A) cells (15 g cdw/L) for the production of (*R*)-MA in a reaction mixture containing KP buffer (200 mM, pH 8.0, 0.5% glucose) and *n-*hexadecane (1:1, v/v) at 30^o^C for 24 h. Benzyl alcohol was used as internal standard (I.S.)


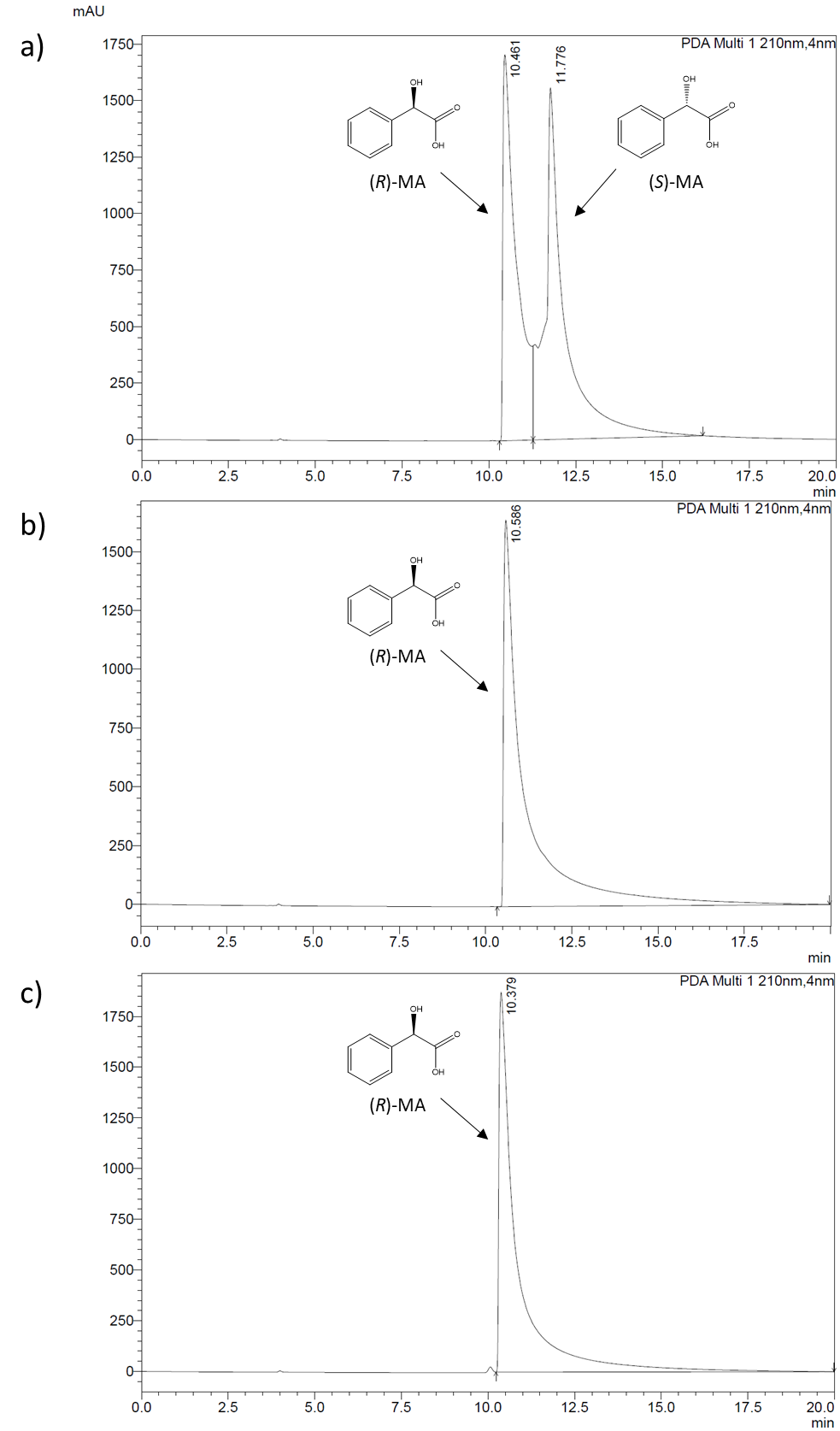


**Fig. S16.** Chiral HPLC chromatograms of (*R*)-MA. a) Racemic MA standard, b) (*R*)-MA standard, c) Isolated (*R*)-MA products from biotransformation of *L*-Phe to (*R*)-MA with *E. coli* (P2S2A) cells.


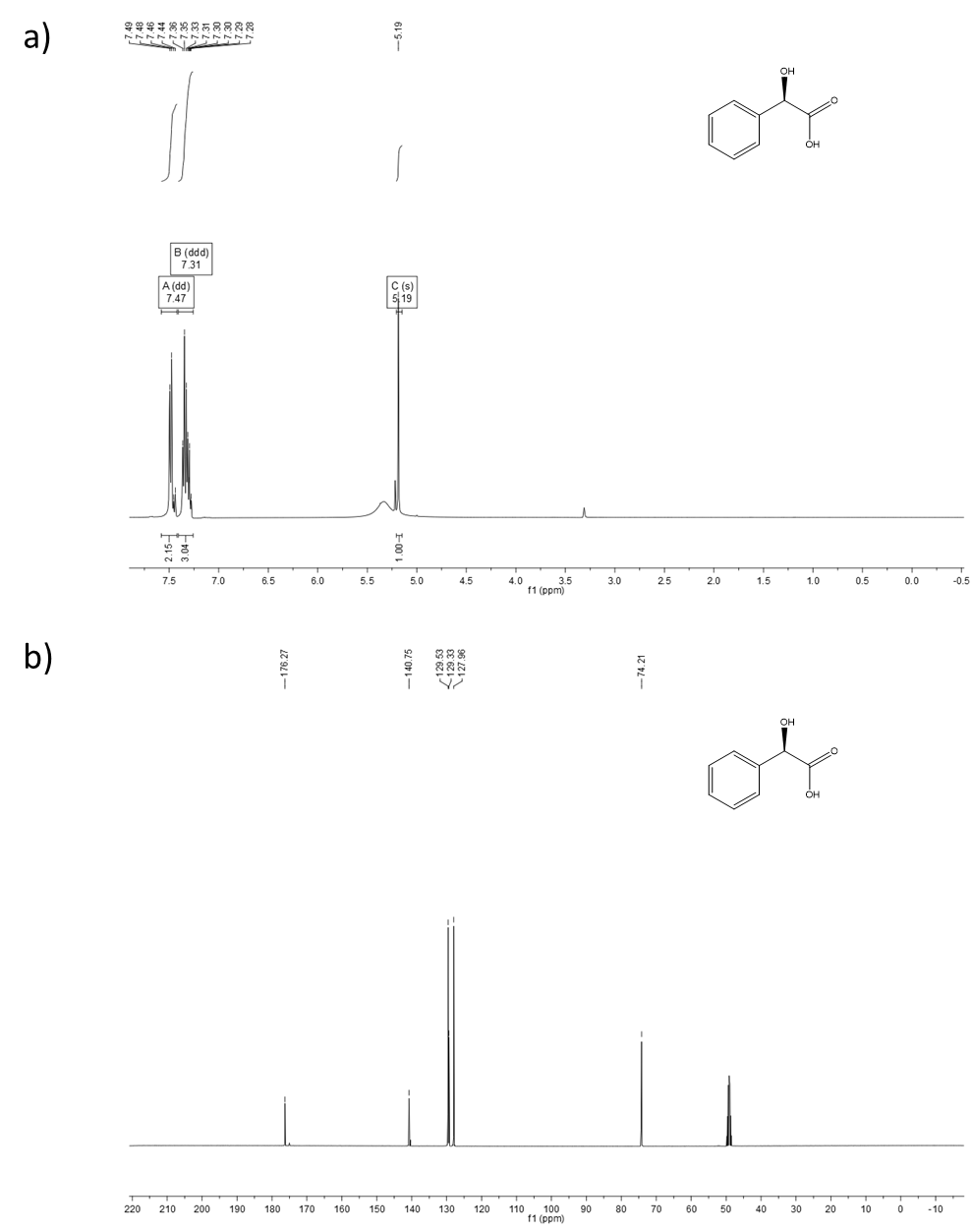


**Fig S17.** NMR spectrum of (*R*)-MA prepared from biotransformation of *L*-Phe with *E. coli* (P2S2A) cells. a) ^1^H NMR spectrum (400 MHz, CD_3_OD), δ 7.47 (dd, *J* = 15.1, 7.2 Hz, 2H), 7.31 (ddd, *J* = 10.7, 9.0, 5.1 Hz, 3H), 5.19 (s, 1H). b) ^13^C NMR spectrum (101 MHz, CD_3_OD), δ 176.27, 140.75, 129.53, 129.33, 127.96, 74.21.

**Fig. S18.** Time course of biotransformation of glucose to (*R*)-MA *via* coupling *E. coli* NST74-Phe and *E. coli* (P2S2A) cells in aqueous-*n-*hexadecane two-phase system


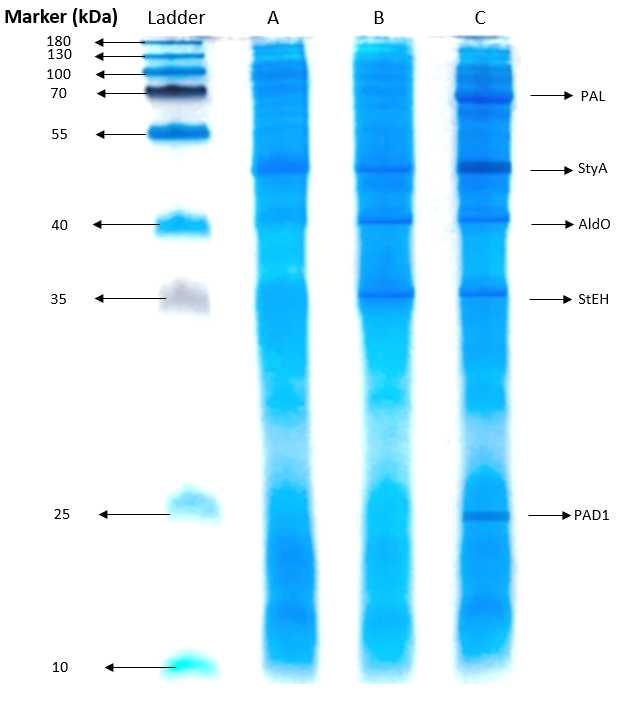


**Fig. S19** SDS-PAGE analysis of total intracellular protein of *E. coli* cells used for the bioproduction of (*R*)-MA; Lane A: *E. coli* (AldO); B: *E. coli* (S2A); C: *E. coli* (P2S2A)

# References

Cochrane FC, Davin LB, Lewis NG (2004) The Arabidopsis phenylalanine ammonia lyase gene family: kinetic characterization of the four PAL isoforms. Phytochemistry 65(11):1557-1564.

Dominic P. H. M. Heuts EWvH, Dick B. Janssen and Marco W. Fraaije (2007) Discovery, characterization, and kinetic analysis of an alditol oxidase from *Streptomyces coelicolor*. J Biol Chem 282(28):20283-20291.

Lindberg D, Gogoll A, Widersten M (2008) Substrate-dependent hysteretic behavior in StEH1-catalyzed hydrolysis of styrene oxide derivatives. The FEBS Journal 275(24):6309-6320.

Panke S, Witholt B, Schmid A, Wubbolts MG (1998) Towards a biocatalyst for (*S*)-styrene oxide production: characterization of the styrene degradation pathway of *Pseudomonas* sp. strain VLB120. Appl Environ Microbiol 64(6):2032-2043.

Payne KAP, White MD, Fisher K, et al. (2015) New cofactor supports α, β-unsaturated acid decarboxylation *via* 1,3-dipolar cycloaddition. Nature 522:497-501.

Sekar BS, Lukito BR, Li Z (2019) Production of natural 2-phenylethanol from glucose or glycerol with coupled *Escherichia coli* strains expressing *L*-phenylalanine biosynthesis pathway and artificial biocascades. ACS Sustain Chem Eng 7:12231-12239.

van Hellemond EW, Vermote L, Koolen W, et al. (2009) Exploring the biocatalytic scope of alditol oxidase from *Streptomyces coelicolor*. Adv Synth Catal 351(10):1523-1530.

Zhou Y, Wu S, Mao J, Li Z (2018) Bioproduction of benzylamine from renewable feedstocks *via* a nine-step artificial enzyme cascade and engineered metabolic pathways. ChemSusChem 11(13):2221-2228.
